# Supplementary material for: Room-Temperature Silicon Platform for GHz-Frequency Nanoelectro-Opto-Mechanical Systems
Source: ACS Photonics. 2022 Feb 1;9(2):413–9. doi: 10.1021/acsphotonics.1c01614 (PMC9523580; doi:10.1021/acsphotonics.1c01614)
Supplement: Supplementary file 1 — ph1c01614_si_001.pdf [file ph1c01614_si_001.pdf]

Supporting information:

Room-temperature silicon platform for GHz-frequency nano-electro-opto-mechanical systems

Daniel Navarro-Urrios[1,2], Martín F. Colombano[1], Guillermo Arregui[1], Guilhem Madiot[1], Alessandro Pitanti[3], Amadeu Griol[4], Tapani Makkonen[5], Jouni Ahopelto[5], Clivia M. Sotomayor-Torres[1,6] and Alejandro Martínez[4]

[1] *Catalan Institute of Nanoscience and Nanotechnology (ICN2), CSIC and BIST, Campus UAB, Bellaterra, 08193 Barcelona, Spain*

[2] *MIND-IN2UB, Departament d'Electrònica, Facultat de Física, Universitat de Barcelona, Martí i Franquès 1, 08028 Barcelona, Spain*

[3] *NEST, Istituto Nanoscienze – CNR and Scuola Normale Superiore, Piazza San Silvestro 12, I-56127, Pisa, Italy*

[4] *Nanophotonics Technology Center, Universitat Politècnica de Valencia, Spain*

[5] *VTT Technical Research Centre of Finland Ltd, P.O. Box 1000, FI-02044 VTT, Espoo, Finland*

[6] *Catalan Institute for Research and Advances Studies ICREA, 08010 Barcelona, Spain*

Device fabrication

All our devices were built on 150 mm silicon wafers on which a 1 μm SiO_2 layer was grown by thermal oxidation. Then, a 220 nm layer of amorphous silicon was deposited by low-pressure chemical vapour deposition (LPCVD). Annealing at temperatures between 650 and 950ºC transformed the silicon from amorphous to nanocrystalline [1]. We have produced various samples in which different annealing temperatures have been applied. This has allowed us to tune the tensile stress of the nc-Si layer, which ultimately resulted in different mechanical properties [2]. This was followed by sputtering 250 nm of aluminium nitride (AlN) on the nc-Si film, followed further by sputtering 100 nm of aluminium to pattern the IDTs. The windows for the NEOM circuits are opened selectively in the AlN layer against the nc-Si using slightly alkaline wet etching. Curved IDTs were patterned using optical lithography, limiting the maximum mechanical frequency to 2 GHz, and dry etching. The OM cavities and the coupling waveguides were defined on the nc-Si window opened between IDTs using electron-beam lithography and dry etching. The cavities and waveguides were finally released by removing the SiO2 layer in HF vapour. A set of more than 10 devices with an equivalent design have been tested, all of them displaying qualitatively similar results in terms of the optical and mechanical properties of the OM nanobeams and in terms of sensitivity and response to the coherent piezo-electrical excitation.

Experimental setup

The experimental setup consists of an optical circuit starting with a tunable laser spanning the range between 1.44 to 1.64 μm connected to a tapered fiber in the shape of a microloop. The polarization state of the light entering the tapered region is set with a polarization controller.

By using a fiber circulator it is possible to collect both the reflected and transmitted signals, which are detected by InGaAs fast photoreceivers PD1 and PD2, respectively. The loop is brought into contact with an integrated waveguide adiabatic coupler [3]. Once the input laser is in optical resonance with the OM cavity, its mechanical motion, activated either by thermal Langevin forces or by the resonant mechanical excitation generated by an IDT, modulates the optical mode spectral position and hence the magnitude of the reflected signal. The RF spectrum of the latter is measured using a spectrum analyzer (SA) with a bandwidth of 13.5 GHz. For coherent piezoelectric excitation and optical detection of mechanical motion we use a vector network analyser (VNA) with port 1 connected to a source IDT and port 2 to the electric output of PD1. In this configuration S_{21} displays the coherent component of the photodetected signal. Throughout this work the laser power is set to be on the order of the hundreds of μW so that radiation pressure forces can be neglected and the optical signal is only used for transducing the mechanical motion.

Piezo-optical coherent response: Noise level, IDT bandwidth, phase response, injected power dependence and IDT dependence.

Figure S1 shows the comparison between the piezo-optical coherent response to an electrical excitation (S_{21}) provided by a Vector Network Analyzer (VNA) when the tunable laser is in and out of resonance with the optical cavity mode at 1560 nm of the OM crystal (black and blue curves respectively). The inset to Figure S1 shows a zoom of the reflection spectrum to illustrate the two optical excitation schemes. The electrical driving power is -10dBm, the sweeping spectral bandwidth goes from 1.9 to 2.2 GHz and the resolution bandwidth is 1kHz. The black curve shows that the bandwidth of the IDTs is about 0.2 GHz and that, away from its bandwidth the S_{21} tends to the experimental noise background. The latter can be estimated in the blue curve to be about -120 dB without any relevant spectral feature. Depending on the quality of the contact between the metal tips and the Al contacts of the IDTs, S_{21} can substantially vary its magnitude for a fixed VNA electrical power.

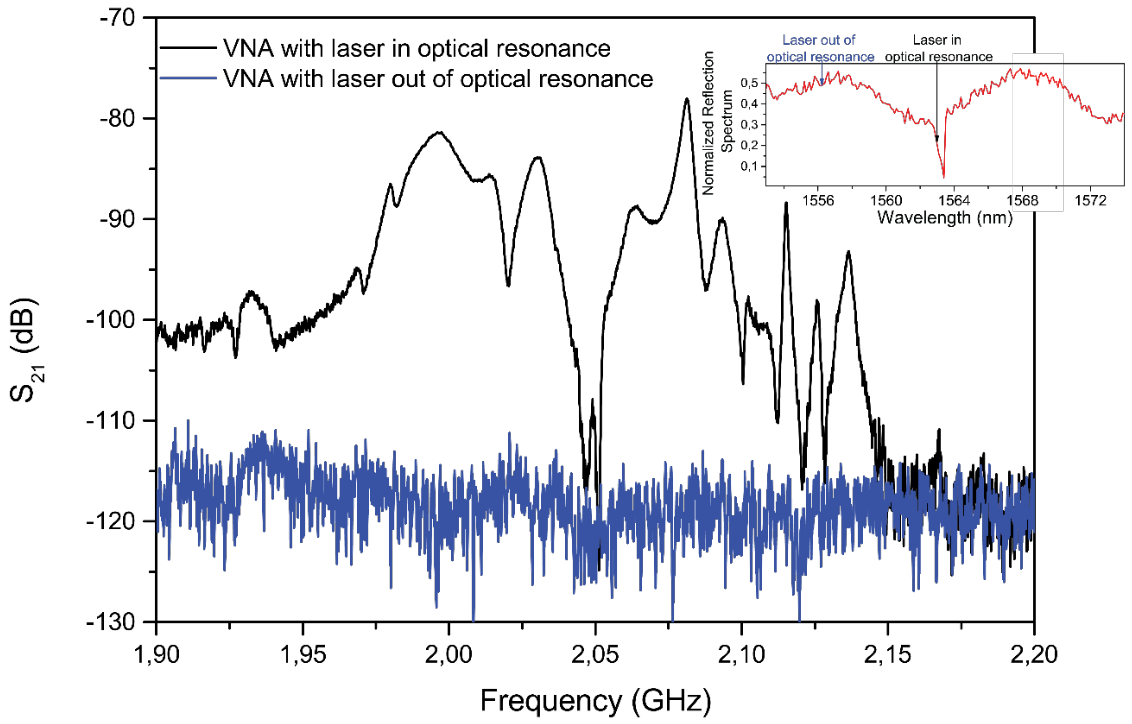

Figure S1. Piezo-optical coherent response when the tunable laser is in and out of resonance with an optical cavity mode of the OM crystal (black and blue curves respectively). The inset shows the reflection spectrum around the optical resonance used to optically probe the system.

Figure S2 shows both the magnitude and phase of the piezo-optical coherent response when the tunable laser is in resonance. The phase is well defined within the bandwidth of the IDT, undergoing, as expected for a driven damped harmonic oscillator, a π shift every time that a mechanical resonance is swept. By averaging the phase signal outside the bandwidth of the IDT it tends to 0 (it averages out).

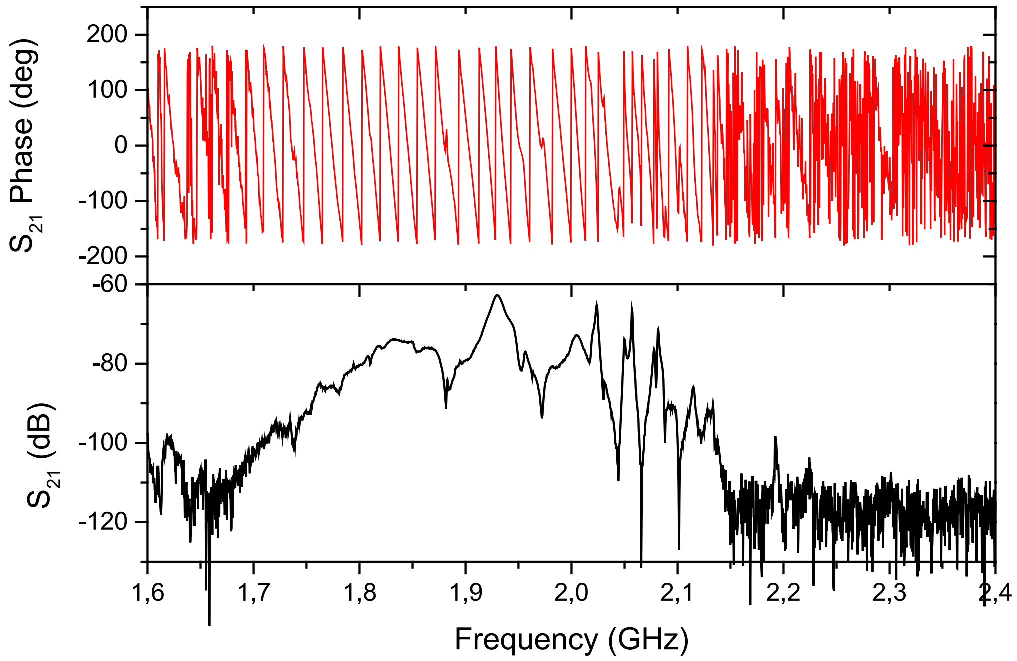

Figure S2. Piezo-optical coherent response when the laser in in resonance with an optical cavity the first observed optical mode. The top panel shows the phase and the bottom panel the magnitude of S_{21}.

Figure S3 shows the S_{21} spectral response as a function of the VNA electrical driving power. We observe that the magnitude of S_{21} associated to the mechanical peaks is constant throughout the studied power range, which allows concluding that the mechanical response of the OM crystal to the IDT excitation is linear with the injected power.

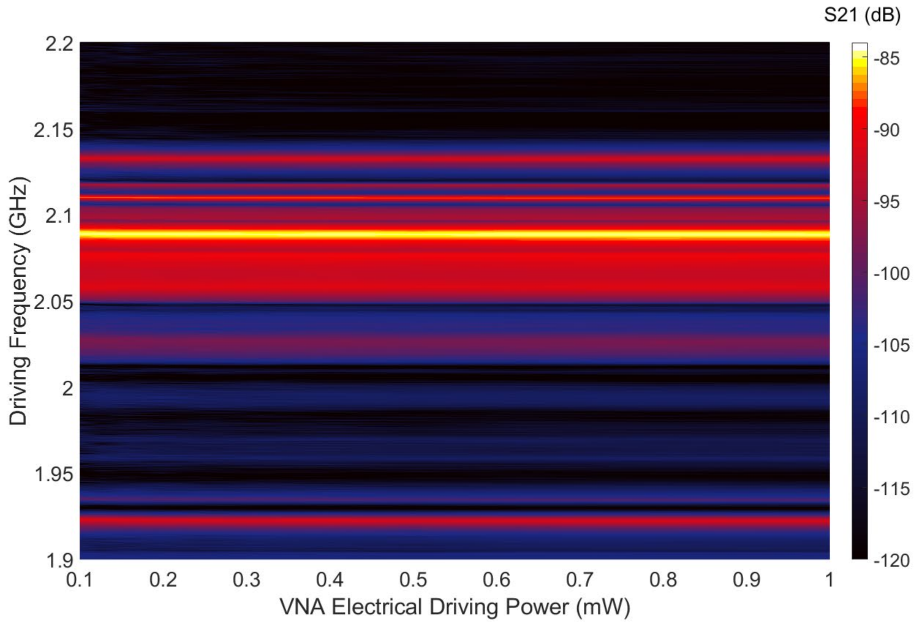

Figure S3. Piezo-optical coherent response when the laser in in resonance with the first observed optical cavity mode as a function of the VNA electrical driving power.

As stated on main text, the piezo-optical coherent response is determined by the OM coupling rate (which depends only on the OM cavity and not on the IDTs), on the spectral response of the IDT and on the efficiency of the mechanical excitation of the specific OM cavity mechanical modes. If all the supported GHz cavity modes were to be excited equivalently by an IDT, the piezo-optical coherent response will be very much alike to the RF spectra observed when the modes are only excited thermally. However, we have observed spectral differences between the coherent RF signals and the thermal RF signal (see Figure 2 of the main text) and between the coherent RF signals obtained with each IDT. In fact, in Figure S4 we show that substantial differences in the peak overall and relative strength appear in S_{21} depending on which of the IDTs is used for SAW excitation. The bandwidth of the IDTs is significantly larger than the frequency range in which the ~2GHz OM cavity modes appear, so we can safely assume that this is not the main cause of the discrepancies. Therefore, we can associate the observed differences to variations on the OM cavity mechanical modes excitation efficiency. Indeed, the latter value strongly depends on the relative positioning between the IDT and the OM cavity. Given that both geometries are processed in a different fabrication step, it is very likely that small relative misalignments can appear, thus impacting in a different piezo-optical coherent response for each IDT.

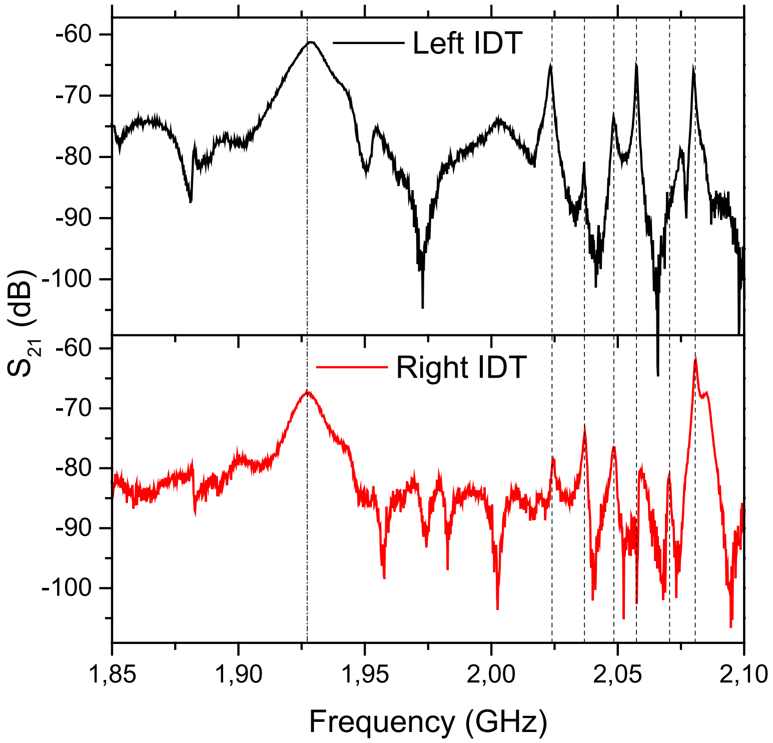

Figure S4. Piezo-optical coherent response when the laser in in resonance with the first observed optical cavity mode and the IDT SAW launcher is the one placed on one side of the OM crystal or the other (top and bottom panels respectively). Notice that some of the OM cavity mechanical modes are highlighted with vertical dashed lines.

RF spectra versus VNA output power

Figure S5 shows the RF spectra measured in the Spectrum Analyzer when a tone at $\Omega_{\mathrm{coh}}/2\pi$=2.058 GHz has been launched in the IDTs and the VNA power has been varied between -40 dBm and 0 dBm. The frequency has been chosen to appear at the centre of the mechanical resonance displaying the strongest transduced signal. The analysis of these curves in terms of the number of coherent phonons that are excited in the OM crystal is showed in Figure 3 of the main text.

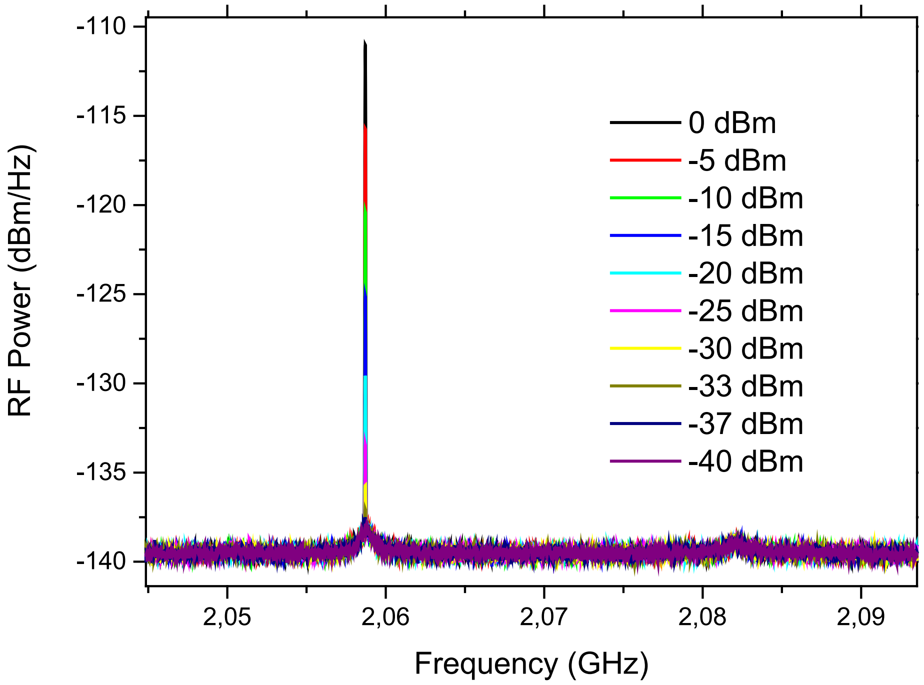

Figure S5. *RF spectra measured in the Spectrum Analyzer versus VNA electrical injection power*

References

[1] D. Navarro-Urrios, N.E. Capuj, J. Maire, M. Colombano, J. Jaramillo-Fernandez, E. Chavez-Angel, L. L. Martin, L. Mercade, A. Griol, A. Martinez, C. M. Sotomayor-Torres, J. Ahopelto, “Nanocrystalline silicon optomechanical cavities,” Opt. Express 26, 9829-9839 (2018).

[2] D. Navarro-Urrios, M. F. Colombano, J. Maire, E. Chavez-Angel, G. Arregui, N. E. Capuj, A. Devos, A. Griol, L. Bellieres, A. Martinez, K. Grigoras, T. Hakkinen, J. Saarilahti, T. Makkonen, C. M. Sotomayor-Torres, J. Ahopelto, "Properties of Nanocrystalline Silicon Probed by Optomechanics", Nanophotonics, 9(16), 4819-4829 (2020)

[3] S. Gröblacher, J. T. Hill, A. H. Safavi-Naeini, J. Chan, and O. Painter, “Highly efficient coupling from an optical fiber to a nanoscale silicon optomechanical cavity,” Applied Physics Letters 103, 181104 (2013).
